# Recent Progress in Vascular Aging: Mechanisms and Its Role in Age-related Diseases

**DOI:** 10.14336/AD.2017.0507

**Published:** 2017-07-21

**Authors:** Xianglai Xu, Brian Wang, Changhong Ren, Jiangnan Hu, David A. Greenberg, Tianxiang Chen, Liping Xie, Kunlin Jin

**Affiliations:** ^1^Zhongshan Hospital, Fudan University, Shanghai 200032, China.; ^2^Department of Pharmacology and Neuroscience, University of North Texas Health Science Center at Fort Worth, TX 76107, USA.; ^3^Department of Urology, the First Affiliated Hospital, Zhejiang University, Zhejiang Province, China.; ^4^Institute of Hypoxia Medicine, Xuanwu Hospital, Capital Medical University. Beijing, China.; ^5^Buck Institute for Research on Aging, Novato, CA 94945, USA.; ^6^Department of Thoracic Surgery, Shanghai Chest Hospital, Shanghai Jiaotong University, Shanghai, China.

**Keywords:** vascular aging, stroke, dementia, arterial stiffness, endothelial dysfunction

## Abstract

As with many age-related diseases including vascular dysfunction, age is considered an independent and crucial risk factor. Complicated alterations of structure and function in the vasculature are linked with aging hence, understanding the underlying mechanisms of age-induced vascular pathophysiological changes holds possibilities for developing clinical diagnostic methods and new therapeutic strategies. Here, we discuss the underlying molecular mediators that could be involved in vascular aging, e.g., the renin-angiotensin system and pro-inflammatory factors, metalloproteinases, calpain-1, monocyte chemoattractant protein-1 (MCP-1) and TGFβ-1 as well as the potential roles of testosterone and estrogen. We then relate all of these to clinical manifestations such as vascular dementia and stroke in addition to reviewing the existing clinical measurements and potential interventions for age-related vascular dysfunction.

## Introduction

Among aging-related diseases, age-specific mortality rates from cardiovascular diseases (CVDs) rise exponentially from more than 40% of all deaths at age 65-74 to almost 60% in the 85 and over age group to become the leading cause of death [1]. The Global Burden of Disease Study reported that 15.6 million people died from CVDs worldwide in 2010, accounting for 29.6% of all deaths, or twice as many as the deaths from cancers [2]. This proportion is even higher in Europe, where CVDs are responsible for nearly half of all deaths [3]. Cerebrovascular diseases, if considered separately, accounts for 12% of all deaths in Europe annually. Moreover, cerebrovascular diseases such as vascular cognitive impairment dramatically decrease the quality of life, even though they do not cause death directly. Therefore, aging research is of great importance, especially in the context of cerebrovascular diseases, from bench to bedside. Understanding the mechanisms underlying age-induced vascular pathophysiological alterations also holds possibilities for developing clinical diagnostic methods and new therapeutic strategies.

The current review will discuss the molecular alterations of aging vessels and their associated clinical conditions, especially in cerebral vessel-related diseases. We will also address possible therapeutic approaches, which may potentially improve cardiovascular and cerebrovascular health in the aged.

## Signaling pathways participating in vascular aging

### Renin/angiotensin system and pro-inflammatory factors

The renin/angiotensin system (RAS) is of great importance in vascular biology, mediating normal vessel function and participating in pathogenesis. Ang II acts through the AT1 and AT2 receptors and Ang II signaling plays a crucial role in the vascular remodeling process [4, 5].

**Figure 1.**
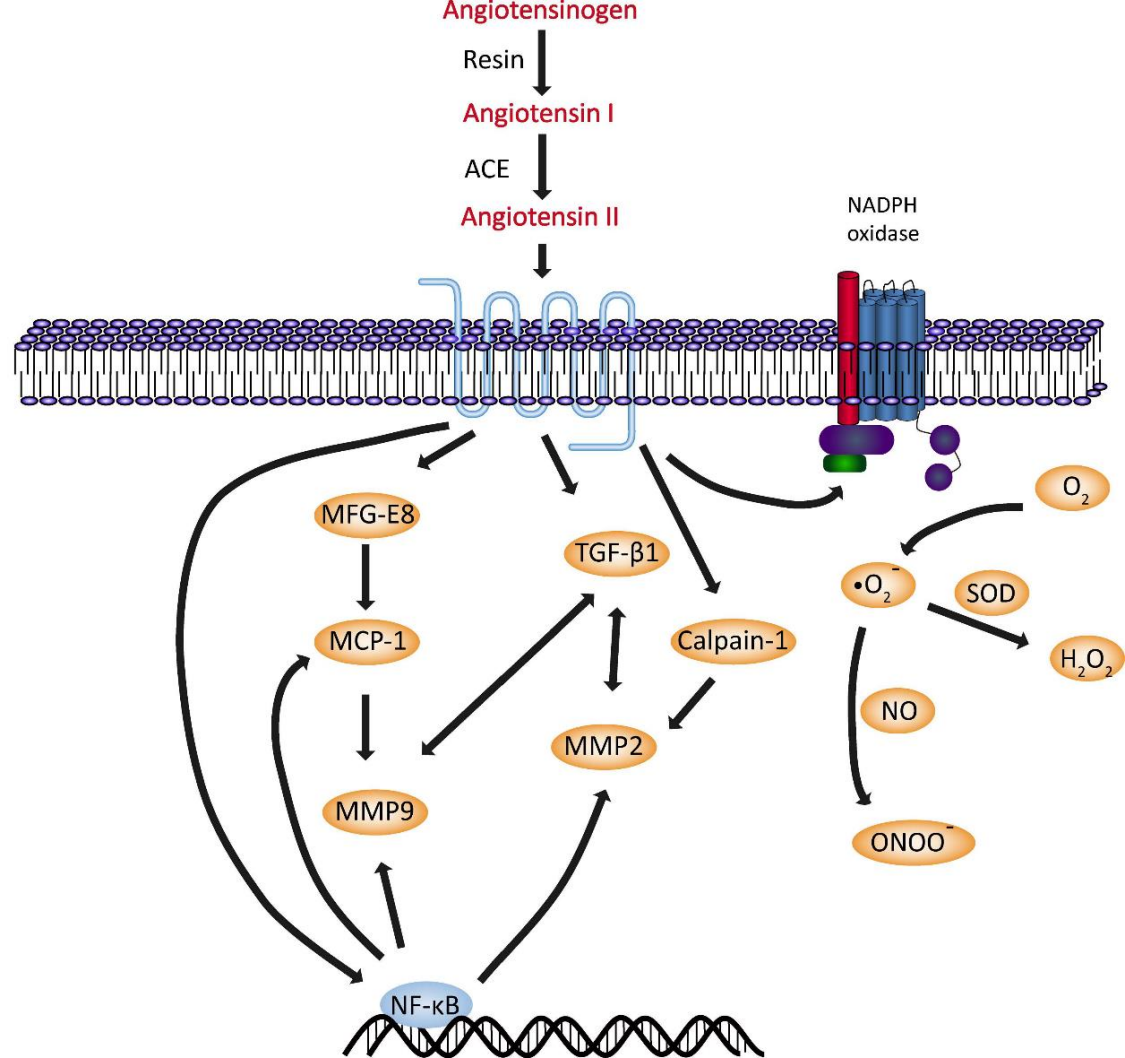
Angiotensin II signaling pathway underlying vascular aging.

The majority of Ang II is the product of Ang I cleavage by angiotensin converting enzyme (ACE). The expression and activity of the angiotensin-converting enzyme-1 (ACE-1) significantly increase in both ECs and VSMCs during aging [6-8]. Chymase, an alternative angiotensin convertase, also shows increased expression within the aging arterial wall [7, 8]. Consequently, the cleaved product, Ang II, is markedly upregulated in rats, monkeys and humans [7-9]. AT1 levels also increase within the aged arterial wall. In contrast, the expression of ACE2 decreases with age, reducing its inhibitory effects on the RAS, which might lead to higher expression levels of Ang II and more Ang II-related vascular alteration [10]. Thus, the net activity of the RAS is increased in the elderly. Under normal physiological conditions, the RAS is well regulated. However, increased activity is associated with several vascular dysfunctions (Fig. 1) [11, 12].

Ang II is an inflammatory trigger that increases the activation of inflammatory factors in the arterial wall with advancing age. It activates nuclear transcription factor-kappa B (NF-κB), a key inflammatory marker, through the AT1 and AT2 receptors and then initiates an inflammatory signaling loop [13]. Induction of NF-κB upregulates the activities of MMP-2/-9, calpain-1, MCP-1, TGF-β1 and ROS, which may contribute to structural and functional vascular remodeling.

Angiotensin II induces dysfunction and ROS in human brain microvascular endothelial cells, promotes cerebrovascular remodeling, vascular inflammation and results in the impairment of regulation of cerebral blood flow [14]. Comparing with placebo, clinical studies have shown that some RAS-inhibiting agents improve arterial stiffness (as evidenced by a reduction in PWV), endothelial dysfunction or number of EPCs independently of blood pressure reduction. Although data relating the RAS activity to these vascular functions are inconclusive, there are more consistent studies that show that drugs interfering with Ang II reduce arterial stiffness and even decrease arterial-stiffness-associated diseases such as cardiovascular, metabolic and renal disorders [15-18].

Moreover, Ang II directly regulates permeability of BBB ECs via oxidative stress [19, 20]. Studies have shown that RAS blockade improves cerebrovascular structure, normalizes CBF autoregulation and reduces cerebral ischemia [21-23] while improving vascular compliance and endothelial function in healthy normotensive elderly individuals [24]. Some evidence indicate that RAS blockade therapy may be effective in the prevention of cognitive decline and dementia [25, 26], which may be independent of its blood pressure lowering function [27]. Drugs that inhibit the RAS include angiotensin converting enzyme inhibitors (ACEI) and angiotensin receptor blockers (ARB). ACEI blocks ACE and decreases Ang II production while ARB blocks the angiotensin type 1 receptor rather than type 2. The RAS is involved in the production and clearance of amyloid-β (Aβ) and vascular and inflammatory factors, which may contribute to AD [28, 29]. Thus, ACEI and ARB may be protective against cognitive impairment in hypertensive patients. However, according to several clinical studies, the effects of RAS blockade on cognitive impairment and dementia have been controversial [30]. A meta-analysis that combined randomized clinical trials and observational studies, suggested that the use of RAS blockade was associated with a 35% reduction in cognitive impairment incidence and 20% reduction in AD incidence. Among RAS blockade, ACEI and ARB have 13% and 31% association with the decrease in the incidence of AD, respectively, while no significant difference was seen between ACEI and ARB in AD risk. RAS blockade provided a significant reduction in the incidence rate of cognitive impairment with age, with ARB being relatively more effective [31].

#### MFG-E8

MFG-E8 is a major component of the human milk fat globule and a multifunctional glycoprotein found in mammary epithelial cells, ECs, VSMCs, dendritic cells, blood cells and fetal hematopoietic stem cells [32-34]. Abundant MFG-E8 expression can be found in advanced human atherosclerotic plaques [35] and certain tumors [36], where it functions in accumulating apoptotic cells, accelerating atherosclerosis and inhibiting neutrophil migration through αVβ3-integrin-dependent MAP kinase activation [37]. MFG-E8 is N-linked glycosylated and is overexpressed in the intima and aortic wall media of aged rodents, nonhuman primates and humans.

Double-label immunofluorescence staining show that MFG-E8 co-localizes with a marker of VSMCs, α-smooth muscle actin. It co-localizes with Ang II and MCP-1 as well and may take part in developmental homeostatic processes, showing protective effects in various models of organ injury [38, 39]. MFG-E8 is known to support both cell-cell and cell-lipid interactions. It also mediates tissue remodeling of different organs including the vasculature, under pathological conditions [39, 40]. Treating early passage VSMCs from the young aorta with Ang II can enhance the expression of both MFG-E8 and MCP-1, which are required for increased VSMC invasion. MFG-E8 siRNA can inhibit Ang II induced MCP-1 protein expression, whereas exposure to MFG-E8 increases MCP-1 expression level and invasion capacity of VSMCs; these effects, in turn, are inhibited by a MCP-1 receptor blocker, the poxvirus-derived viral CC-chemokine inhibitor protein, vCCI. Conversely, knockdown of MFG-E8 by siRNA also substantially reduces VSMCs invasion capacity. These data suggest that Ang II is upstream of MFG-E8 while MCP-1 is a downstream molecule within the Ang II signaling cascade that regulates VSMC invasion. MFG-E8 is a pivotal relay element within the Ang II/MCP-1/VSMC invasion signaling cascade [40].

MFG-E8 affects VSMC proliferation as well. Exogenously overexpressing MFG-E8 in young VSMCs increases their proliferation rate. Treatment with MFG-E8 triggers a dose-dependent activation of ERK1/2, which are important modulators of mitosis, in both young and aged VSMCs. Exposure to MFG-E8 also enhances levels of PCNA and CDK4, which can be blocked by the ERK1/2 phosphorylation inhibitor, U126, and promotes VSMC proliferation via αVβ5 integrins. In contrast, knocking down MFG-E8 by stealth siRNA dramatically reduces PCNA and CDK4 levels and slows down proliferation [41]. Proliferation of VSMC can be regulated by PDGF and its receptor-signaling cascade as well [42]. Expression of the PDGF receptor-α and PDGF receptor-β is increased in aged VSMCs. MFG-E8 treatment dose- and time-dependently increases expressions of PDGF-A, PDGFR-α and PDGFR-β, in young and old VSMCs while MFG-E8 siRNA decreases expressions of PDGFR-α and PDGFR-β [41]. Collectively, an increase in MFG-E8 signaling may be a mechanism underlying the age-associated increase in aortic VSMC proliferation and invasion.

Endothelial integrity is of great importance to a normal vessel. However, aging enhances endothelial cell (EC) susceptibility to apoptosis. The overexpression of MFG-E8 in ECs triggers apoptosis through increasing the Bax/Bcl-2 ratio and caspase-9 and caspase-3 activation. Knocking down MFG-E8 by siRNA decreases both caspase-3 activity and the phosphorylation of glycogen synthase kinase 3β, thereby suppressing advanced glycation end product (AGE)-induced apoptosis [43].

#### MMPs

The extracellular matrix (ECM) maintains vascular structural stability and is essential for the mechanical and biological properties of vessel walls. The ECM is degraded with advancing age, which facilitates VSMC migration.

MEROPS (https://merops.sanger.ac.uk/), an online peptidases database, classifies metalloprotease families into 15 different clans such as MA, MC and 7 unassigned families. MMPs comprise the M10 family of the zinc-containing MA clan of metallopeptidases. They are ubiquitous enzymes, with an active site where a Zn atom, coordinated by three histidines, can play a catalytic role to degrade the vascular ECM [44]. MMPs involved in vascular ECM remodeling consist of collagenases (MMP-1 and MMP-8, expressed by ECs and SMCs), gelatinases (MMP-2, expressed by SMCs and macrophages; MMP-9, expressed in macrophages and vascular cells), elastases (MMP-7, expressed at a low level in the vascular wall, MMP-12, synthesized by macrophages), and stromelysins and membrane-type metalloproteinases (MT-MMPs) [45-47]. In addition, MMP-8 can promote the migration and proliferation of VSMCs [47].

Among the family of MMPs, increased MMP-2 and MMP-9 expressions and activities were found to be associated with enhanced Ang II signaling of aged arteries in various species including humans [8, 9, 48, 49]. MMP-2 activation is increased during aging, localized to ECs and VSMCs and was detected in the intima, internal elastic lamina and elastin fibers of the inner tunica media [50]. MMP-9 activity is increased *in situ* during aging in grossly normal aortic segments. Increased MMP-9 levels reduce vascular permeability and induce inflammation, which leads to vessel and perivascular pathophysiological alterations. Additionally, chronic cerebral hypoperfusion associated with vascular aging upregulates MMP-2 expression in microglia and the vascular endothelium of white matter [51].

#### Calpain-1

Calpain-1 is a ubiquitous cytosolic Ca^2+^-activated neutral protease. As discussed in above, increased MMP-2 can be found in aging vessels and MMP-2 activation is implicated in age-associated VSMC migration, elastin degradation and collagen deposition. Calpain-1 regulates MMP-2 activity in VSMCs, facilitating vascular calcification and fibrosis [48] and promoting the invasion of fibroblasts and leukemic cells [52].

Ang II signaling mediates the age-related increase in MMP-2 activity in the vascular wall. This can be blocked using an exogenous or endogenous calpain inhibitor (calpastatin), indicating that calpain activity is required for Ang II-associated activation of MMP-2 [48]. Calpain-1 is also linked to cytoskeleton remodeling within VSMCs and VSMC migration as migratory capacity can be stimulated by overexpression of calpain-1 in young VSMCs. Furthermore, calpain-1 has higher mRNA transcript and protein abundance and activity in the aged rat aorta and is regulated by Ang II signaling. Collectively, these findings indicate that increased calpain-1 activity is a central mechanism for the exaggerated Ang II signaling involved in age-related arterial remodeling.

#### MCP-1

MCP-1 is a 76-amino acid member of the C-C subfamily of chemokines [53]. Through the activation of CCR2, a 7-transmembrane G protein-coupled receptor, MCP-1 can promote the migration of monocytes, lymphocytes, ECs, VSMCs and human fibroblasts as well as induce MMP-1 synthesis [54-56]. MCP-1 also mediates TGF-β-induced angiogenesis by stimulating VSMC migration. The upregulated expression of MCP-1 and CCR2 could be found in the intima of the aorta of aged rats [57]. MCP-1 expression by vascular cells is regulated by Ang II via the NF-κB-dependent pathway and Ang II levels are upregulated with aging [58, 59]. Interestingly, an age-dependent increase of circulating MCP-1 levels was found in healthy aged people without known diseases. MCP-1 plasma level is increased in older individuals. This means that MCP-1 has the potential to serve as an aging biomarker, although its specific biological significance and functions still need to be further investigated.

#### TGF-β1

TGF-β1 is a widely expressed cytokine, playing a critical role in the processes of proliferation, wound healing, synthesis of ECM molecules and inflammation. It also has multiple functions in both vascular development and remodeling [60].

TGF-β1 is synthesized as a latent precursor molecule (LTGF-β) that contains an amino-terminal hydrophobic signal peptide region, named the latency-associated peptide (LAP). LAP is cleaved from the complex in the Golgi apparatus, but remains non-covalently bonded with TGF-β1. LTGF-β is secreted as a small latent complex (SLC) or as a large latent complex that is covalently bonded with the LTGF-β binding protein (LTBP) [60]. Inflammatory stimuli, MMP-2 and MMP-9 promote the release of TGF-β1 from this complex in its mature form, which binds to its receptor and then activates downstream signaling pathways.

The active receptor complex phosphorylates the receptor-activated Smads (R-Smads), Smad2 and Smad3, which propagate the signal. R-Smads form complexes with Smad4 and translocate to the nucleus, where they recruit co-activators or co-repressors into transcriptional complexes and regulate the transcriptional activity of various genes [61]. The signal transduction pathway has its own endogenous regulators as well. An inhibitory Smad, Smad7, blocks TGF-β1 signaling by physically interacting with the activated TGFBR1 receptor and prevents the phosphorylation of Smad2/3 [62].

Importantly, upregulated Ang II and MMP2/MMP9 increase the activation of TGF-β1 [63, 64]. Signaling molecules downstream of activated TGF-β1 (p-Smad-2/-3/-4) are increased while inhibitory Smad (Smad-7) is decreased in the arterial wall with aging [65]. TGF-β1 signaling affects ECs function and causes arterial stiffening by increasing collagen types I and III in vessel walls [66, 67].

#### ROS and NO bioavailability

Oxidative stress is widely acknowledged as an important factor associated with aging and disease, which increases in the arterial system of humans and experimental models. It has been linked to the development of age-related pathogenesis, leading to arterial dysfunction. Reactive oxygen species also play a physiological role in the vascular wall, including endothelium-dependent functions, smooth muscle and endothelial cell growth and survival, and regulation of remodeling of the vascular wall [68]. Importantly, oxidative stress can be altered by the imbalance between antioxidant defenses and reactive oxygen species (ROS) that are produced in vessel walls and regulate cell functions and cellular senescence [69]. Imbalance in the regulation of oxidative stress contributes to vascular alterations characterized by mitochondrial dysfunction and increased ROS production, and, eventually, leads to the development of cardiovascular pathological alterations, such as hypertension and stroke.

Ang II can enhance oxidative stress as well as impair endothelial function [70]. Increased Ang II signaling activity can promote the generation of cellular ROS, together with the activation of redox-sensitive signaling cascades [71]. Therefore, ROS may be essential signaling molecules for maintaining vascular homeostasis.

Superoxide molecules are produced by transferring an electron to oxygen; superoxide concentrations can be reduced to the picomolar levels by superoxide dismutase (SOD). However, superoxide molecules react with nitric oxide (NO) at least 10 times faster than SOD can scavenge NO [72]. This reaction may have some biological significance when the concentration of superoxide molecules rises in blood vessels with advancing age [73, 74]. The higher level of superoxide inhibits the generation of NO by vascular cells, resulting in the impairment of endothelium-dependent relaxation. On the other hand, both eNOS activation and NO bioavailability are decreased with age [75, 76]; lower NO levels further increase ROS production. Moreover, peroxynitrite (ONOO^-^), a highly reactive molecule produced by the reaction of NO with superoxide, has been implicated in impaired EC function and vascular aging [74]. Several studies reported the importance of age-related mitochondrial oxidative stress as a characteristic of endothelial dysfunction. It is associated with the over-activation of NADPH oxidase, an enzyme localized in the cytoplasm and membranes. The NADPH oxidases are considered to be enzymatic sources of ROS production in vascular cells, while over-activated NADPH oxidase mediated ROS production is regulated by various factors including hypoperfusion and cytokines and hormones such as Ang II, platelet-derived growth factor, TGF-β as well. ROS promote age-related vascular dysfunction not only by compromising EC function, but also inducing the expression of factors involved in vascular structural remodeling such as MMPs [77, 78].

Mitochondrial DNA (mtDNA) is a double-stranded DNA circular genome that encodes 37 genes including tRNA, rRNA and respiratory chain enzymes. MtDNA has a higher mutation rate than nuclear DNA due to the lack of protective histones and the close proximity of the mitochondrial genome to the inner membrane where ROS are continually generated. The amount of mtDNA mutations have been observed to increase as humans age [79] e.g., increased deletions in mtDNA have been reported in the aged human central nervous system and cardiac muscle [80-82]. Mutations of mtDNA may accumulate as a result of unrepaired DNA damage caused by ROS or by replication errors that undergo clonal expansion throughtout the adult life [83]. The replication of mtDNA occurs randomly and independently of the cell cycle [84]. Furthermore, a single cell may contain more than one type of mutated mtDNA, namely heteroplasmy. Once it reaches a certain threshold, respiratory chain dysfunction may occur.

Mitochondria oxidative stress increases with age and is a strong trigger of age-related ECs and VSMCs function. Antioxidant enzymes knockout animal models with ablated mitochondrial aldehyde dehydrogenase or mitochondrial superoxide dismutase showed increased mitochondrial ROS formation and oxidative mtDNA lesions. Meanwhile, endothelium-dependent acetylcholine-induced relaxation of aortas was significantly deteriorated. Due to the correlation analysis, mitochondrial ROS formation is directly related to endothelial function while the correlation between mtDNA damage and endothelial dysfunction is relatively less striking but still efficient [85]. Studies showed that H_2_O_2_ is able to induce mtDNA damage in ECs and VSMCs. H_2_O_2_ treatment leads to mtDNA damage within 10 and 15 minutes in ECs and VSMCs, respectively. Apart from H_2_O_2_, either low or high ONOO^-^ concentration causes mtDNA damage in ECs while only high ONOO^-^ concentration in VSMCs causes mtDNA damage, indicating that ECs are more sensitive to ROS than VSMCs [86]. A mouse model with a homozygous mutation in the exonuclease encoding domain of the mtDNA polymerase gamma (Polg^m/m^) that is prone to age-dependent accumulation of mtDNA mutations was reported to have increased mitochondrial ROS levels, which resulted in an accelerated form of age-dependent cardiomyopathy. Oxidative stress and respiratory chain dysfunction due to mtDNA point mutations accumulation in protein-coding mitochondrial genes are considered to contribute to the premature aging phenotype of the Polg^m/m^ mice. The susceptibility of mtDNA to oxidative damage may be considered a combination of factors besides the higher superoxide formation rate in the mitochondrial matrix, which is different from nuclear DNA, in that the mtDNA has no protective histones and possesses a low level of DNA repair activity. Therefore, the mitochondrial 8-oxo-deoxyguanosine DNA lesion may be the underlying burden of oxidative stress during the aging process in the heart and brain [87].

Human clinical studies have shown that the mtDNA^4977^ deletion levels in the putamen, cortex and cerebellum accumulates with advancing age. The levels of mtDNA damage in the cortex and putamen of the aged brain is much higher than the aged heart [81]. Numerous studies indicated that mtDNA deletion is related to neurodegenerative disorders such as Alzheimer’s disease, Parkinson’s disease, Huntington’s disease and amyotrophic lateral sclerosis [88-91].

Furthermore, mtDNA from atherosclerotic plaques or circulating cells from CVDs patients show increased frequency of mtDNA adducts or the common mitochondrial deletion mtDNA^4977^, compared with normal vessels or patients without CVDs, respectively [92, 93]. However, the direct correlation between mtDNA damage and neurological diseases via vascular aging remains unclear.

### Sex hormones

#### Testosterone

The majority of testosterone is produced by the testis while small amounts are produced by the adrenal glands. Testosterone plays an essential role in the development of the reproductive system, promoting male secondary sexual characteristics.

Testosterone can freely diffuse across the plasma membrane, enter the cytoplasm and bind to the androgen receptor (AR). The hormone-bound AR acts as a transcription regulatory element that binds to specific DNA response elements in target gene promoters, thereby modulating transcription and protein synthesis [94]. Simultaneously, there are other non-classical and non-genomic mechanisms of testosterone action. Testosterone can activate intracellular signaling molecules such as the mitogen-activated protein kinase (MAPK) family, ERK1/2, protein kinase A and protein kinase C pathways [95]. ARs are widely distributed in cells and organs including ECs and VSMCs [96]. Thus, testosterone levels may influence vascular function in health and disease.

Circulating testosterone in men declines progressively with aging, starting in the early years of adulthood. The decline is paralleled by a number of pathophysiological alterations leading to cardiovascular and cerebrovascular diseases, metabolic syndrome and insulin resistance [97]. Testosterone is involved in vasorelaxation via endothelium-dependent mechanisms, ion channel modulation, and vascular structural remodeling.

Testosterone can modulate endothelial function through NO release [98]. Physiological concentration of testosterone increases NO synthesis by ECs through rapid recruitment of the ERK1/2 and PI3-kinase/Akt signaling pathways [99, 100]. Moreover, testosterone stimulates NO synthesis, which increases cyclic guanosine monophosphate (cGMP) formation in VSMCs to induce vasorelaxation [101]. A low plasma testosterone level is associated with endothelial dysfunction in men independent of other risk factors, suggesting a protective effect of testosterone on the endothelium [102]. In addition, testosterone is metabolized by aromatase to form 17β-estradiol, which stimulates NO release.

Testosterone also modulates ion fluxes in VSMCs by either activating K^+^ channel or inactivating Ca^2+^ channel. Testosterone within the physiological range is a selective and potent inhibitor of L-type voltage-operated Ca^2+^ channels, which results in vasodilation [103]. Testosterone also interacts with a large conductance voltage- and Ca^2+^-activated K^+^ (BK_Ca_ or MaxiK) channel, inducing vascular relaxation by increasing intracellular K^+^ efflux [104]. This channel regulates vascular tone and diameter and is a target for various vasoconstrictor and vasodilator agents under physiological conditions. Its expression is reduced in aged coronary arteries, suggesting decreased vasodilating capacity [105]. Long-term testosterone deprivation in rat VSMCs reduces expression of the Kv1.5 voltage-gated K^+^ channel protein whilst restoration of testosterone to physiological concentrations rescues the resulting impairment in Kv1.5 channel function [106]. Intimal-medial thickness and PWV can be reduced by long-term testosterone administration [107, 108], indicating that testosterone replacement can diminish vascular remodeling and arterial stiffness associated with male hypogonadism [109]. Furthermore, low testosterone levels give rise to higher responsiveness to vasoconstrictor agents [104, 110].

Testosterone induces relaxation of isolated blood vessels *in vitro*. Testosterone levels in men are positively associated with flow-mediated dilatation (FMD) as an independent determinant of endothelial vasomotor function in men [102]. Short-term intracoronary testosterone administration has been reported to induce vasodilation that can increase blood flow in men [111]. A supraphysiological concentration of testosterone was able to trigger direct vasodilator actions via membrane ion channels in isolated blood vessels rather than through the endothelium and AR-dependent pathway [112]. On the other hand, oral supplementation of testosterone in men enhanced FMD without affecting the basal diameter of the brachial artery, suggesting improved endothelial function in men [113]. Progressively lower concentrations of total or free testosterone would be associated with increasing incidence of cerebrovascular events such as incident stroke and transient ischemic attack [114]. However, the relationship between low testosterone level and AD is not well understood. A meta-analysis of seven prospective cohort studies with a total of 5251 elderly men and 240 cases of Alzheimer’s disease, supports the view that low testosterone level is significantly associated with increased risk of AD in elderly men, which may suggest that testosterone has important roles in regulating cognitive function in elderly men [115]. Nevertheless, although low testosterone level is a risk factor for AD, a long-term testosterone administration study of 36 months in older men with low or low-to-normal testosterone concentrations treatment did not improve cognitive function [116]. This may indicate that testosterone treatment could be used as maintenance therapy in that it lowers the risk of AD, but does not necessarily improve brain function. In addition, Jamadar et al. (117) reviewed 11 clinical studies examining the effects of androgen-deprivation therapy (ADT), a mainstay therapy for advanced prostate cancer (PCa), on cognition as measured by standardized tests in cognitive domains. They found that spatial memory and verbal memory may be especially sensitive to ADT, indicating that it is important to consider the benefits of ADT, especially in patients with early stage prostate cancer, which might contribute to vulnerability to negative cognitive effects.

#### Estrogen

Estrogen is a protective factor for the cardiovascular and cerebrovascular systems [118]. Both types of estrogen receptors (ERs), ERα and ERβ, can be found in the vascular endothelium, SMCs and adventitia. Like testosterone, estrogens bind to nuclear receptors. Activation of ERs regulates gene expression through estrogen-response elements to mediate transcription. Estrogen can also bind to plasma membrane sites thus inhibiting Ca^2+^ L-type voltage-gated channels in VSMCs and activating several signaling pathways including the PI3K pathway in ECs [119, 120].

Estrogen is known to mediate vascular function by increasing NO bioavailability and suppressing ROS levels. Estrogen modulates vasoconstrictive factors or positively upregulates vasodilating factors such as prostacyclin (PGI_2_). Estrogen also inhibits the expression or function of cyclooxygenase-derived vasoconstrictors including prostaglandin H2 and thromboxane A2, which are mediators of vascular tone in females [121]. Furthermore, ACE activity can be decreased by estrogen therapy, which reduces Ang II production, seen in both animal models and postmenopausal females. Moreover, estrogen attenuates AT1 receptor expression and AT1-mediated responses in the aorta, heart, and kidneys [122].

In middle-aged women, subclinical vascular dysfunction may be aggravated by estrogen deficiency due to menopause or ovariectomy, which may be improved by estrogen replacement. However, some evidence suggest that estrogen can delay the progression of atherosclerotic lesions, measured by carotid artery intima-media thickness, in early postmenopausal women [123]. Beneficial effects of estrogen replacement therapy on mood and cognition in early but not late postmenopausal women have also been reported [124, 125]. Further research into sex hormone deficiency related to vascular dysfunction is still necessary to define optimal diagnostic and therapeutic strategies.

The menstrual cycle may be a great estrogen fluctuation cycle for studying the function of estrogen on vascular function. A comprehensive study reported that FMD, acetylcholine administration to skin microvessels via iontophoresis and arterial compliance, were all consistently reduced during the early luteal phase, while pulse wave velocity did not [126, 127], suggesting that vascular endothelial function possibly fluctuates with hormonal changes throughout the menstrual cycle although there was little alteration found in arterial stiffness. However, longer periods of estrogen deficiency may trigger changes in arterial stiffness. Several studies have reported the augmenting age-related increase in arterial stiffness in postmenopausal women [128-131]. Moreover, the evidence is inconclusive on the relationship between menopause and cognition. Some suggest that menopause at younger ages was associated with reduced cognitive performance in later years [132, 133], while others reported that no clinically significant changes were found with respect to cognitive function [134, 135] or that only short-lasting cognitive decline was observed in limited domains with menopause [136, 137].

## Diseases linked to vascular aging

Blood pressure changes with advancing age. There is a linear rise in systolic blood pressure induced by arterial stiffness and a concurrent increase in diastolic blood pressure due to endothelial dysfunction-induced high peripheral vascular resistance until about the age of 50 [138]. Thereafter, high systolic blood pressure accelerates arterial stiffness, perpetuating a vicious cycle. In older individuals, increased arterial stiffness decreases diastolic blood pressure while systolic blood pressure keeps rising, which forms an age-related systolic-diastolic blood pressure divergence, resulting in a widen pulse pressure. Even in older normotensives, they have higher arterial stiffness, systolic blood pressure and pulse pressure *vs.* the young [139]. Increasing pulse pressure or hypertension increases cardiac afterload, then leads to left ventricular hypertrophy. In the brain, increased pulse pressure penetrates further into cerebral microcirculation, which may potentially expose capillaries to damaging levels of elevated pressure pulsatility. Thus, cerebral microvascular remodeling and dysfunction increase vascular resistance in response to elevated pulse pressure and limit the penetration of excessive pulsatility directly into the capillaries [140]. However, cerebral microvascular remodeling and dysfunction increases minimal resistance and reduces vasodilatory reserve. Accompanying age-related microvessel loss, these lead to an increased susceptibility to hypoperfusion and reduce the efficiency of oxygen and energy delivery, which can promote cerebral microvascular disease [141, 142] and cognitive impairment [143, 144].

Thus, vascular aging may be linked with neurological diseases in two parallel pathways: (1) reduction in brain microcirculation, arterial stiffening, endothelial dysfunction causing hypoperfusion and (2) blood-brain barrier breakdown associated with brain accumulation of serum proteins and several vasculotoxic and/or neurotoxic macromolecules ultimately leading to secondary neuronal degenerative changes.

### Alzheimer’s disease and vascular cognitive impairment

Dementia is a disorder of cognitive impairment that interferes with everyday life. Alzheimer’s disease (AD) is the most common cause of dementia and is rare before 60 years of age [145]; vascular cognitive impairment (VCI) is a group of cognitive disorders with a presumed vascular cause and has generally superseded the term vascular dementia. In addition to AD and VCI’s adverse effects on affected individuals and their families, dementia greatly increases demands on the medical care system. VCI is a larger concept that includes VCI-no dementia, vascular dementia (VD) and cognitive impairment of mixed origin (Alzheimer’s disease and vascular dementia) [146]. In this section, we focus on vascular-related cognitive impairment by reviewing recent studies.

Several reports have shown that vascular aging is associated with both AD and VD. Hanon et al. [147] observed that PWV was elevated in AD (13.3±2.9 m/s) and VD (15.2±3.9 m/s) patients compared with controls (11.5±2.0 m/s), suggesting that arterial stiffness may be involved in the development of these disorders. Calik, et al. [148] pointed out that arterial diastolic dysfunction and increased arterial stiffness are detected in AD patients. Other studies have demonstrated that vascular stiffness is an independent risk factor for and has potential to be a strong predictor of cognitive loss [149, 150]. Interestingly, PWV is significantly higher in VD than in AD [151], which has a more complex etiology in which the contribution of vascular disease is less clear. Endothelial dysfunction may associate with cognitive impairment in the elderly population as well. It was reported that mildly cognitive impaired patients with prevalent amnestic multiple domains exhibited much worse brachial FMD [152].

Arterial stiffness is largely associated with Aβ deposition, either by baseline comparison or accumulating in the brain over the years in non-demented elderly adults; this association differs with different arterial bed measurements [153, 154]. Peripheral rather than central vascular stiffness is relatively stronger in the correlation with the amount of Aβ being deposited whilst systemic arterial stiffness (a composite parameter of central and peripheral arteries) or central arterial stiffness is strongly correlated with the progression of Aβ accumulation. Furthermore, as a result of arterial stiffness and endothelial dysfunction, pulse pressure increases and becomes a risk factor for AD and dementia in older adults [155]. High pulse pressure promotes vascular remodeling, which further impairs vascular function. Clinical data showed that increased pulse pressure is related to reduced cerebrospinal fluid Aβ, indicating that elevated pulse pressure may affect the clearance of Aβ [156]. This was confirmed by another group that pulse pressure elevation was significantly associated with reduced cerebrospinal fluid Aβ in the aged group [157].

An alternative mechanism may involve reduced cerebral blood flow (CBF). Decreased CBF was observed by arterial spin-labeling MRI in the frontal and parietal cortices of human subjects with either AD or VD [158]. Total (tCBF) and mean CBF decrease with age whereas pulsatile CBF velocity increases [159], perhaps due to the age-related increase in stiffness of elastic arteries and decreased compliance of muscular arteries and arterioles. Interestingly, females have relatively higher tCBF and lower cerebrovascular resistance than males [160]. *In vivo* studies demonstrated that subsequent hypoperfusion accelerates deposition of Aβ via increased generation and impaired clearance and could cause cognitive impairment [142, 161]. Greater Aβ concentration decreases the production of NO, thus causing a significant decrease in endothelial-dependent vasodilation along with the induction oendothelial cell apoptosis [162-164].

### Stroke

Stroke is the leading cause of death worldwide that accounts for about 1 in 10 deaths or 5.7 million deaths a year. Stroke incidence is doubled in low- and middle-income regions compared with those living in high-income regions [165]. It can be fatal or dramatically decrease the quality of life in survivors and often necessitates extremely costly medical care. Stroke incidence increases with advancing age, so research on the relationship between vascular aging and stroke could have significant clinical benefits.

Arterial stiffness is an independent predictor for fatal stroke as demonstrated in a long-term clinical study of 1715 patients with essential hypertension whose carotid-femoral PWV was measured at entry. Importantly, the predictive value of PWV remained significant even after full adjustment for classical cardiovascular risk factors such as age, gender, cholesterol level, diabetes and smoking [166]. Another study comprising 2835 participants in the third examination phase of the Rotterdam Study, demonstrated that arterial stiffness is an independent predictor of stroke among otherwise healthy subjects. During follow-up, 63 subjects developed a stroke (mean follow-up period, 3.2 years). Among this group, the risk of stroke increased with increasing aortic PWV index. Although other comorbidities of aging such as obesity and diabetes limit the predictive value of the conventional cardiovascular risk factors, PWV may be an independent predictor of coronary heart disease and stroke [167]. Willum-Hansen, et al. [168] also documented the prognostic value of PWV in a 1678-person population-based study. The meta-analysis consisted 10 studies that revealed that carotid stiffness is strongly associated with a higher stroke incidence, independent of aortic stiffness, supporting the idea that carotid stiffening is an important factor in the pathogenesis of stroke [169]. Indeed, the increased pulse wave is able to go through the carotid artery quickly and penetrate distally into the cerebral microcirculation, which may promote hypertrophic remodeling and induce chronic ischemia by hypoperfusion.

Arterial stiffness also serves as a predictor of stroke outcome. Low carotid-femoral PWV is significantly associated with excellent stroke outcome, even after adjustment for age, baseline NIHSS score on admission and stroke history [170]. In stroke patients, aortic PWV helps predict asymptomatic coronary artery disease beyond the predictive ability of classical risk factors. In fact, coronary artery disease is a significant cause of morbidity and mortality in patients who have had a stroke [171]. Further, arterial stiffness is independently related to cerebral microbleeds in patients with stroke [172].

Endothelial dysfunction is another potentially adverse alteration with advancing age in stroke development. Endothelial dysfunction reduces the tone of aging arteries and increases their sensitivity to vasoconstrictors or stimulus, which may result in a higher risk of acute or chronic ischemia. It has been reported that acute ischemic stroke patients have severe endothelial dysfunction [173]. More specifically, the relationship between endothelial dysfunction and stroke subtypes has been studied, which revealed that only lacunar stroke is closely related with endothelial dysfunction [174]. Endothelial dysfunction may be involved in the pathogenesis of lacunar stroke especially in those subjects with concomitant silent lacunar infarcts and ischemic white matter hyperintensities (WMH) [175]. Endothelial function measured by brachial FMD is impaired in stroke patients compared with healthy controls, which has also been observed in other studies [176, 177]. Selecting homocysteine, von Willebrand factor (vWF), E-selectin, P-selectin, intercellular adhesion molecule-1 (ICAM), and vascular cellular adhesion molecule-1 (VCAM) as blood markers of endothelial dysfunction, Wiseman et al. [178] found that these markers were significantly increased in lacunar stroke *vs.* the non-stroke group. On the cerebrovascular level, by assessing cerebral vasoreactivity and extracerebral vascular functions in symptomatic lacunar stroke patients and non-stroke controls, symptomatic lacunar stroke patients had more severe endothelial dysfunction in the cerebral arteries while relatively smaller difference of extracerebral arterial abnormalities were found. Thus, endothelial dysfunction of brain vessels can be an important determinant of lacunar stroke [179]. Overall, vascular aging may have important roles in stroke pathogenesis and could be a potential marker of stroke risk and outcome.

**Table 1:** Devices and methods used for evaluating arterial stiffness.

Device	Manufacturer	Method	Region or arterial site
**Regional arterial stiffness**			
Sphygmocor	AtCor Medical, Au	Pressure	cfPWV
Diasys	Novacor, Fr	Korotkov sounds & ECG	baPWV
Omron VP-1000	Omron Healthcare, Jp	Pressure	cfPWV, baPWV, faPWV
Vasera system	Fukuda Denshi, Jp	Pressure & ECG	Cardio-ankle vascular index
Mobil-O-Graph	I.E.M. Ge	Pressure	cfPWV
PulsePen	Diatechne, It	Pressure & ECG	cfPWV
PulseTrace	Micromedical, UK	Doppler & ECG	cfPWV, baPWV
Vicorder	SMT medical GmbH & Co, Ge	Pressure	cfPWV, baPWV
pOpmètre	Axelife SAS, Fr	Photodiodes sensors	Finger-toe PWV
**Local arterial stiffness**			
Artlab system	Esaote, It	Ultrasound	CCA, CFA, BA
E-Tracking	Aloka, Jp	Ultrasound	CCA, CFA, BA
MRI	-	Cine-MRI	Aorta

ECG, electrocardiography; CCA, common carotid artery; CFA, common femoral artery; BA, branchial artery

### Other neurological diseases

Cerebral small vessel disease (SVD) is common in aged people and can be potentiated by hypertension and diabetes mellitus [180]. SVD can be visualized by MRI as WMH, lacunar infarcts, and cerebral microbleeds [181]; it also contributes to the development of cognitive decline and dementia [182, 183]. Several studies have reported an association between arterial stiffness (measured based on brachial-ankle or carotid-femoral pulse wave velocity) and SVD [184-187]. Age is associated with WMH, although the relationship between age and lacunar infarction, in particular, differs among studies. Some studies report no statistically significant link [188, 189], but this may be explained by the relatively young population (mean age 46.9-58.2 years) and the low prevalence of lacunar infracts (4.3%). When older populations were examined, arterial stiffness was shown to be associated with lacunar infarcts [185, 190-192]. Brain atrophy is also related to vascular aging [193, 194]. Increased aorta or carotid stiffness is associated with larger WMH and increased carotid stiffness is associated with decreased total brain parenchyma, grey matter and especially, white matter volumes. These associations were even stronger in uncontrolled hypertension and aged patients [195].

Taken together, vascular aging may affect the process and outcome of neurological diseases including dementia, cognitive impairment and stoke. Therefore, it needs to be carefully considered as an important detrimental factor in these diseases, especially in certain populations such as patients with hypertension.

Considering the importance of the cerebral blood supply for brain function, SVD, thus, has a great impact on cognitive function. The vascular alterations-induced vascular dysfunction that finally leads to cognitive impairment are diverse, which includes hemodynamic changes or alterations on cerebral blood vessels. General hypoperfusion can produce transient or permanent cognitive impairment [196], which is a result of structural and functional age-related changes. The most acknowledged vascular lesions promoting vascular cognitive impairment is SVD, especially in white matter [197]. These lesions often coexist in one single patient and contribute to the cognitive impairment. Lacunar infarcts and leukoaraiosis that are common in VCI are also associated with arterial stiffness. Patients with leukoaraiosis have higher PWV that transmits increased pulse pressure into the brain through the middle cerebral artery [198]. Tortuous arterioles, increased intima-media thickness, reduced capillaries number and length may be the underlying vascular alterations. Microbleeds is a predictor of cognitive dysfunction and arterial stiffness may be important in the development of microbleeds, especially in stroke patients [172, 199]. Furthermore, it is well known that Aβ deposition or cerebral amyloid angiopathy is associated with vascular cognitive impairment, although it seems to be a “marker” of AD, with it being present in over 90% of patients [200]; it can be promoted by arterial aging-induced arterial stiffness and endothelial dysfunction. AD-related cerebrovascular lesions and VCIs are similar. Data containing different average prevalence of leukoaraiosis and lacunar infarcts, microbleeds, microinfarcts, Aβ deposition as well as hypoperfusion in AD and VCI or VD could reveal the pattern of vascular alterations in these diseases. A previous review reported that the most remarkable vascular-related difference between AD and VCI is cerebral amyloid angiopathy and microinfarcts/lacunes [200]. Cerebral amyloid angiopathy can be found in almost 98% of AD patients *vs.* 30% of VCI patients and 23-45% of aged controls. Microinfarcts/lacunes were seen in 70% of VCI patients *vs.* 10-15% of AD patients and just over 10% of aged controls.

**Table 2:** Approaches for assessing endothelial function.

Method	Vascular site	Invasive or Non-invasive
Coronary epicardial vasoreactivity	Coronary	Invasive
FMD	Peripheral conduit artery	Non-invasive
EndoPAT	Small arteries and finger microvasculature	Non-invasive
Intracoronary doppler wire	Coronary microcirculation	Invasive
Venous occlusion plethysmography	Forearm vein	Invasive

## Clinical investigation of vascular aging

### Measurements

There are many methods that can be used for assessing vascular aging. As vascular aging is a ubiquitous phenomenon with both clinical and subclinical manifestations, large-scale application of invasive techniques may be inappropriate and can lead to overtreatment.

Arterial stiffness can be assessed at different levels, either regional or local. For regional arterial stiffness assessment, PWV seems to be a widely used and reproducible approach. Central arterial stiffness can only be estimated by PWV between the common carotid artery (CCA) and the common femoral artery (cfPWV). cfPWV is often thought to be the gold standard for measuring arterial stiffness as it evaluates the arteries from the aortic through the aortoiliac pathway including big branches, which are essential in the hemodynamic load for the end organ [201]. The femoral-ankle PWV (faPWV) is considered a peripheral arterial stiffness index. Furthermore, brachial-ankle (baPWV) assesses the mechanical property of a large territory, integrating both the large-sized central elastic and medium-sized peripheral muscular arteries thus correlating better with left ventricular mass and diastolic function and other indices of arterial function than cf-PWV; it also can possibly be considered a relative systemic index of arterial stiffness [202]. For local arterial stiffness, superficial arteries can be directly measured by ultrasound. The carotid may be a region with great interest to clinicians not only because it is a superficial artery, but it also is a high incidence region of atherosclerosis. All types of classic bi-dimensional vascular ultrasound devices can probably be used to examine parameters including local arterial distensibility, diameter and thickness. However, because of the limitation of ultrasound technology itself, the readings from devices always differ from clinician to clinician, even with the same device. Thus, there is increasing interest in measuring arterial stiffness through MRI. Interestingly, faster and more robust MRI sequences have enabled MRI-based PWV measurements. Nonetheless, the lack of compatible computing software limited the use of MRI-based PWV [203]. Because of its ease and wide clinical utility coupled with a much lower cost, the ultrasound technology is still the most prevalent method for local arterial stiffness assessment (Table 1).

Endothelial dysfunction is a pathological condition during aging. Similar to measurements for arterial stiffness, endothelial function assessment would require stimulating the human body by pacing or exercise, or stimulating the arteries locally by acetylcholine or reactive hyperemia, then investigate if the blood flow or dilation increases as healthy arteries are expected to. Despite its high cost and invasive process, coronary epicardial vasoreactivity is the gold standard for epicardial macrovascular endothelial dysfunction (Table 2). However, for an asymptomatic individual, non-invasive and cheaper measurements would be appropriate for screening. As a noninvasive approach, FMD has been the most commonly used measurement of endothelial function of the brachial or other peripheral conduit artery. It measures the artery’s response to endothelial NO during reactive hyperemia after a 5-minute occlusion of the brachial artery using a blood pressure cuff to estimates endothelial function. Measuring endothelial function with peripheral arterial tonometry (EndoPAT) is another non-invasive approach that evaluates changes in finger arterial pulse wave amplitude under the stimulation of reactive hyperemia. The contralateral arm could serve as an internal control [204].

**Table 3:** Potential intervention measures for vascular aging.

Intervention Measure	Influence of intervention	Underlying Mechanism	Reference
Exercise	Decreases vessel tortuosity, cardiovascular morbidity and mortality Increases CBF, capillary vascularity Maintain endothelial function	eNOS↑ ROS↓	[205-207]
Caloric restriction	Decreases serum cholesterol, arterial stiffness and attenuate vascular inflammation Protects endothelial function	eNOS↑ ROS↓ Circulating C-reactive protein (CRP) ↓ TNFα↓	[208, 209]
Statins	Decreases PWV Protects endothelial function Anti-vascular inflammation	eNOS↑ Serum CRP ↓ Activity of NF-kB↓ ROS↓	[210-212]
RAS Drugs	Decreases PWV Increases CBF	Block Ang II signaling	[15, 213, 214]

### Potential intervention measures

As life expectancy is expected to increase with the use of modern medical technologies, biomedical research is likely to focus increasingly on cerebrovascular and cardiovascular diseases. Consequently, numerous research groups consider vascular aging an important area of research (Table 3). However, even in the case of less invasive strategies such as exercise and diet, purported benefits of new interventions must be weighed against potential adverse effects.

## Summary

The molecular targets through which aging perturbs vascular homeostasis are manifold and appear to include increased expression or activation of TNF-α, IL-1β, IL-6 family members and CRP, which promote inflammation and endothelial dysfunction. Currently, there are many non-invasive ways to examine vascular aging through arterial stiffness and endothelial function assessments. New interventions such as the use of statins and RAS drugs to treat vascular aging have been suggested, but clinicians and patients should consider potential adverse effects.

What may seem like an insurmountable task with regard to the treatment for vascular dysfunction may in fact hold much promise and excitement. It will take a concerted effort from researchers and clinicians to work together to advance the field of vascular aging and find interventions to promote cardiovascular and cerebrovascular health in the elderly.
